# IChem: A Versatile Toolkit for Detecting, Comparing, and Predicting Protein–Ligand Interactions

**DOI:** 10.1002/cmdc.201700505

**Published:** 2017-11-07

**Authors:** Franck Da Silva, Jeremy Desaphy, Didier Rognan

**Affiliations:** ^1^ Laboratoire d'Innovation Thérapeutique UMR 7200 CNRS—Université de Strasbourg 74 route du Rhin 67400 Illkirch France; ^2^ Discovery Chemistry Research and Technologies Eli Lilly and Company Lilly Corporate Center Indianapolis IN 46285 USA

**Keywords:** cavity, drug design, fingerprints, interactions, virtual screening

## Abstract

Structure‐based ligand design requires an exact description of the topology of molecular entities under scrutiny. IChem is a software package that reflects the many contributions of our research group in this area over the last decade. It facilitates and automates many tasks (e.g., ligand/cofactor atom typing, identification of key water molecules) usually left to the modeler's choice. It therefore permits the detection of molecular interactions between two molecules in a very precise and flexible manner. Moreover, IChem enables the conversion of intricate three‐dimensional (3D) molecular objects into simple representations (fingerprints, graphs) that facilitate knowledge acquisition at very high throughput. The toolkit is an ideal companion for setting up and performing many structure‐based design computations.

IChem is a suite of tools consisting of about 50 000 lines of computer code written in C++, decomposed in nine modules, for detecting and comparing molecular objects (proteins, ligand‐binding cavities, ligands, protein–ligand and protein–protein complexes) frequently manipulated in structure‐based computational chemistry (Table 1). Herein we describe four of the most frequent and important uses. For deeper investigation of all modules, the reader should refer to the publicly available user guide.^1^

**Table 1:** IChem modules.

Module	Purpose
pdbconv	Post‐process raw PDB files
realign	Structural alignment of two molecules
sims	Fingerprint comparison
utils	Miscellaneous
Volsite	Cavity detection and druggability prediction
IFP	Fingerprinting protein–ligand interactions
Ints	Fingerprinting protein–ligand interaction patterns
Grim	Converts protein–ligand interaction pattern in graphs
DetectPPI	predict biologically relevant PPIs^[a]^

[a] Protein–protein interfaces.

**Setting the scene: pdbconv**. A reasonable start to any structure‐based design project is the retrieval of experimentally determined protein structures from the Protein Data Bank (PDB),^2^ a web resource that currently stores over 134 000 entries. Unfortunately, PDB structures cannot be used directly, as many important features (e.g., protonation and ionization states, atom types and bond orders for organic molecules) are missing. The *pdbconv* module of the IChem toolkit automates the preparation of ready‐to‐use protein–ligand structures. The process first assigns a specific class to each residue name (Table 2).

**Table 2:** HET residue classes in IChem, exemplified by one residue.

HET^[a]^	Template^[b]^	Updated^[c]^	Class^[d]^
ACO	1	/	COFACTOR
3NI	1	/	ION
001	1	/	LIGAND
3CO	1	/	METAL
004	1	/	MOD_AA
02I	1	/	NUCLEIC
01L	1	/	ORGANOMET
BH1	0	BPH	PROSTHETIC
ALA	1	/	STD_AA
045	1	/	SUGAR
000	1	/	UNWANTED
HOH	1	/	WATER

[a] Three‐character alphanumeric code of each chemical component. [b] Template present (1) or absent (0). [c] Updated (/) or deprecated and replaced (e.g., BH1 updated into BPH). [d] Residue class.

It then applies a correct atom type to every heavy atom, generates the corresponding covalent bonds, and selects strongly bound water molecules while removing bulk water. The process relies on a predefined list of all possible residues with the corresponding templates for every HET record of the PDB file. Correct atom types and 3D coordinates are provided for every template by converting, with Corina,^3^ PDB SMILES strings into the corresponding MOL2 file.

The residue list (Table 2) assigns the encountered HET record to one of the 12 possible residue classes (cofactor, ion, ligand, metal, modified amino acid, nucleic acid, organometallic, prosthetic, standard amino acid, sugar, unwanted, water). Please note that molecules originating from crystallization buffer (“unwanted” class) are automatically identified and discarded. Once atom types have been properly defined for every molecule type (protein and accessory molecules, solvent, ligand), any third‐party tool (e.g., Protoss)^4^ can be used to finally add the missing hydrogen atoms while optimizing both the ionization and tautomeric state of each molecule of the PDB entry.

Working with predefined residue lists and molecular templates provides both advantages and drawbacks. The main advantage is a uniform treatment of all chemical components of a PDB entry with a presumably correct atom typing. As a main drawback, the procedure requires an updated residue list and thus fails in case of a newly released PDB entry. We therefore propose regular updates along every new release of the in‐house developed sc‐PDB database of druggable protein–ligand complexes.^5^

**Detecting ligandable cavities: Volsite**. *Volsite* is a tool to automatically detect cavities at the surface of a macromolecule of interest, and predict its structural druggability.^6^ It can be run in two modes depending on whether coordinates of a bound ligand are given (ligand‐restricted mode) or not (full unrestricted mode). In any case, the target is first placed in a 2 Å resolution grid lattice and each voxel is assigned a state as whether its accessibility exceeds a user‐defined threshold. Accessible voxels are then assigned a pharmacophoric property (hydrophobic, aromatic, hydrogen bond donor, hydrogen bond acceptor, positive ionizable, negative ionizable) complementary to that of the nearest protein atom according to a set of topological rules.^7^ The pharmacophoric properties of all atoms are detected on the fly by the general IChem atom parser thereby enabling to consider accessory molecules (Table 2) or not during the cavity detection. Because every voxel has a fixed volume, the total number of pharmacophore‐annotated voxels approximates the overall cavity volume. The method is fast (a few seconds) and precisely delineates the cavity borders at a very high precision (Figure 1).

**Figure 1:**
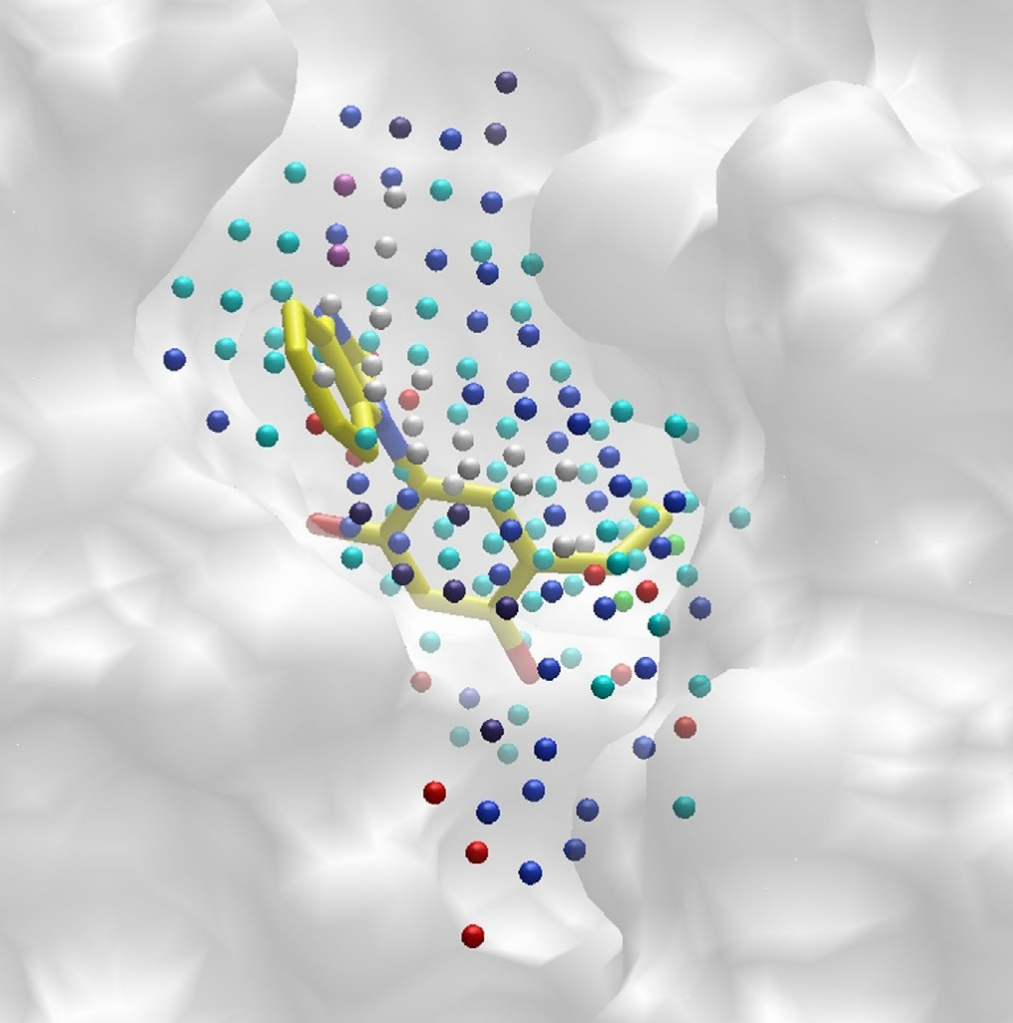
Cavity detection at the surface of the heat shock protein HSP90α (PDB ID: http://www.rcsb.org/pdb/explore/explore.do?structureId=4YKR). The Volsite cavity (volume=502.87 Å^3^, druggability=1.11) is represented by pharmacophoric points (hydrophobic, cyan; aromatic, green; hydrogen‐bond donor, blue; hydrogen bond acceptor, red; positive ionizable, dark slate blue; negative ionizable, orchid; dummy, white). Volsite points nicely encompass the bound inhibitor (HET code 4ep, yellow sticks) ignored during the cavity detection procedure.

In addition, a set of 73 cavity descriptors are computed for each cavity and used as input to a support vector machine (SVM) classifier to predict the structural druggability (or ligandability) of the inspected cavity. In a standard benchmarking exercise consisting of 113 cavities of known druggability, Volsite presented the highest accuracy when compared with state‐of‐the‐art tools.^6^ In case of multiple cavities, all druggable cavities are saved as readable MOL2 files, along with their predicted druggability score.

Interestingly, the similarity of two Volsite cavities can be estimated by analogy to classical ligand similarity measurements, using a companion tool (*Shaper*)^6^ that uses a smooth Gaussian function to maximize the overlap of their volume and pharmacophoric properties. High‐throughput cavity comparisons are increasingly used in computational chemistry notably to identify ligands for novel cavities, design inhibitors with precise selectivity patterns and predict their possible side effects.^8^

**Converting protein–ligand complexes into fingerprints and graphs: IFP, GRIM**. A major feature of IChem is the possibility to generate diverse simplified representations (fingerprints, graphs) of protein–ligand interactions. For example, the *IFP* module enables to list all protein–ligand interactions occurring in a complex and to output an interaction fingerprint as a bit string (Figure 2).

**Figure 2:**
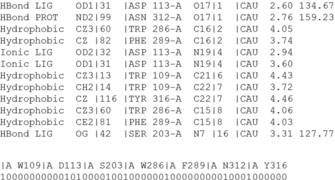
Table of protein–ligand interactions and interaction fingerprint (IFP) generated by IChem. For every ligand‐binding residue, seven bits are switched either on (1) or off (0) as whether a particular interaction is detected or not with the ligand. Interactions are registered in a precise order (hydrophobic, aromatic face‐to‐face, aromatic edge‐to‐face, hydrogen bond accepted by ligand, hydrogen bond donated by ligand, ionic bond with ligand negatively charged, ionic bond with ligand positively charged).

Several years ago, we^7^ and other groups^9^ proposed the use of IFPs to post‐process docking data and pick poses producing IFPs similar to that of known actives. Computing interaction fingerprints (IFPs) from docking poses is a robust and very efficient manner to predict ligand binding modes,^10^ propose reliable scaffold hops,^11^ and enrich virtual hits in true actives.^12^ The success of this post‐processing approach is based on the idea that true ligands of a same target often share key interactions with key anchoring residues and thereby produce relatively similar IFPs. However, a clear limitation is the strict dependence to the number of active site residues, preventing to compare interaction fingerprints across binding sites of different sizes.

We therefore recently designed size‐invariants descriptors conceptualized by a graph describing the exact protein–ligand interaction pattern.^13^ The method called GRIM (Graph Interaction Matching) defines three interaction pseudoatoms (IPAs) for every detected protein–ligand interaction: one on the ligand‐interacting atom, one on the protein‐interacting atom and one at the barycenter of the latter two atoms (Figure 3). The full set of IPAs defines an interaction pattern that is unique to every protein–ligand complex and that can be converted into a graph where IPAs will define nodes.^13^

**Figure 3:**
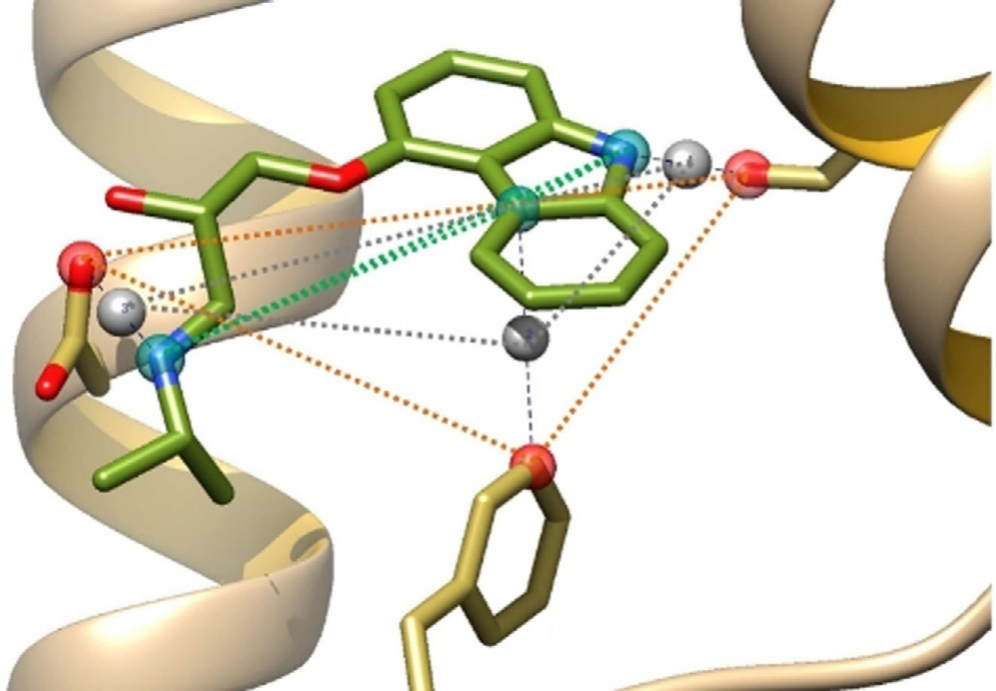
Example of a set of interaction pseudoatoms (ligand‐based, red; protein‐based, cyan; centered, gray) for the β2 adrenergic receptor in complex with carazolol (PDB ID: http://www.rcsb.org/pdb/explore/explore.do?structureId=2RH1).

A particular interaction pattern can be easily compared and aligned to another one by a simple graph matching technique aiming at identifying the maximal common subgraph (clique).^13^ The similarity of two interaction pattern graphs is measured by an empirically derived score (GRIMscore) that can be used for example to post‐process docking poses and reward those corresponding to interaction patterns already visited in reference X‐ray structures (Figure 4). In three consecutive international docking contests aimed at predicting ligand binding modes prior to the release of the corresponding X‐ray structures, GRIM rescoring was always quoted as one of the very best methods for generating near‐native docking poses.^14^ The same advantage over fast scoring functions was reported in virtual screening against diverse target families (e.g., G protein‐coupled receptors, nuclear hormone receptors, protein kinases).^13^

**Figure 4:**
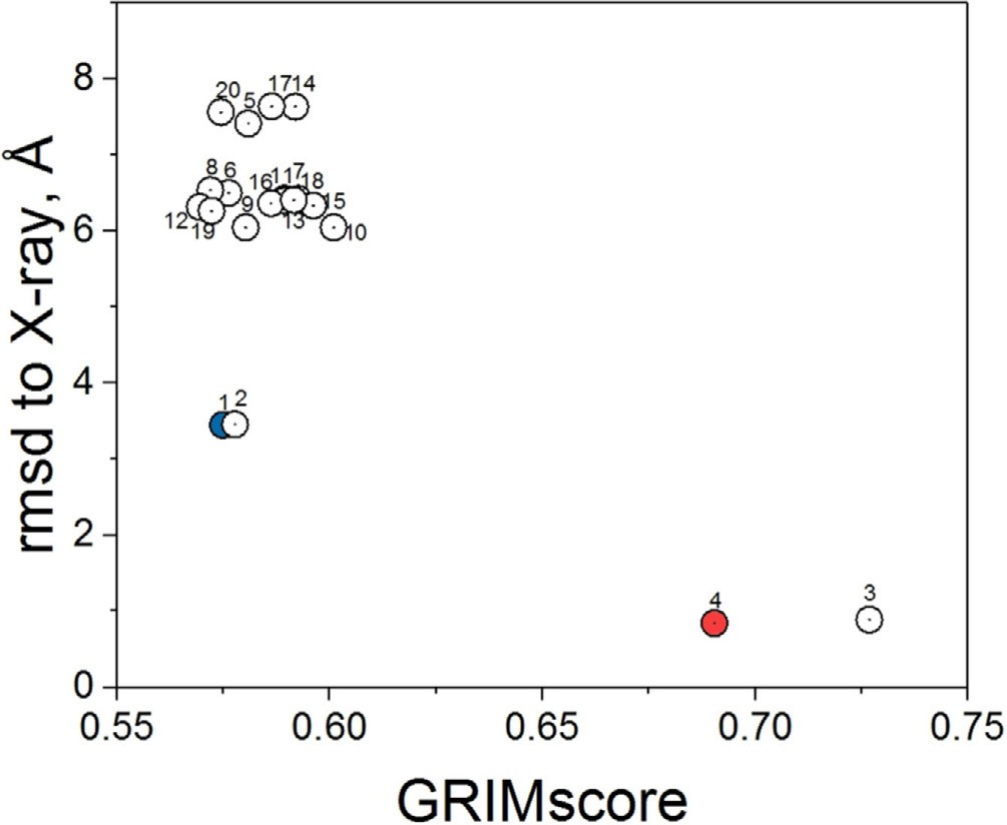
Surflex‐Dock rescoring 20 docking poses of the MAP27 inhibitor14b to the human MAP4K4 by graph similarity (GRIMscore) to the X‐ray structure of the same kinase with the inhibitor GNE‐495 (PDB ID: http://www.rcsb.org/pdb/explore/explore.do?structureId=4ZK5). The top‐ranked pose according to Surflex‐Dock (cyan circle) is irrelevant. The two best poses according to GRIM (red circles) are <1.0 Å RMSD away from the true X‐ray pose. All poses are numbered from 1 to 20 according to the Surflex‐Dock score.

GRIM presents several advantages over alternative knowledge‐based rescoring strategies: 1) it can be coupled to any docking algorithm, 2) it does not constrain ligand docking but rewards interaction patterns already present among PDB templates, 3) it takes advantage of ligands with similar binding modes and not necessarily similar chemical structures, 4) it can be applied in a target family‐biased pose selection process in which PDB templates from the same protein but also from similar targets can be used to store reference interaction patterns, and 5) it permits to directly quantify binding mode similarity between a predicted protein–ligand complex and any PDB template at a very high throughput.

**Detecting biologically relevant protein–protein interfaces: detectPPI**. Protein–protein interfaces (PPIs) represent challenging but very promising targets for drug discovery.^15^ Hence, PPIs describe a vast unexplored biological space for which small molecular weight modulators^16^ are expected to offer very high potency and selectivity profiles. Although mainly discovered by biophysics‐driven fragment‐based approaches, computational chemistry is expected to play a major role in designing the future PPI modulators,^17^ notably upon relying on the huge structural information already available in the Protein Data Bank. To discriminate biologically relevant from crystallographic artifacts, computational methods are needed to rapidly detect PPIs and predict their biological relevance from a structural point of view. IChemPIC^18^ was designed to address this need. The *detectPPI* module uses the general IChem functions (molecule reader, interaction detection) to detect the interface, identifies the corresponding IPAs and generate a fixed‐length property vector (Figure 5) as input for a Random Forest classifier previously trained on a set of 400 PPIs (200 biologically relevant, 200 irrelevant interfaces). IChemPIC is equally robust to detect both classes with the same accuracy, independently on the size of the PPI.^18^

**Figure 5:**
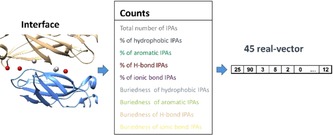
Prediction of biologically relevant protein–protein interfaces by IChem. Interaction pseudoatoms (IPAs) are first computed from the 3D structure of the protein–protein complex and converted into a 45 real vector, featuring important physicochemical properties (interface size, pharmacophoric properties, buriedness), read by a Random Forest classifier for relevance prediction.

Interestingly, this new IChem module can be used at a high throughout to detect biologically relevant PPIs at the PDB scale. Alternatively, the method can be used on‐line (http://bioinfo-pharma.u-strasbg.fr/IChemPIC) by just specifying the PDB three‐letter code.

In conclusion, IChem is a suite of software dedicated to the analysis and comparison of three‐dimensional molecular objects. It converts an intricate three‐dimensional information into much simpler fingerprints or graphs, thereby enabling high‐throughput comparisons and fueling machine learning models for predicting important features like protein–protein interfaces, druggable cavities, interaction patterns and binding poses. IChem is available for nonprofit academic research at http://bioinfo-pharma.u-strasbg.fr/labwebsite/download.html.

## Conflict of interest

*The authors declare no conflict of interest*.
